# Direct conversion of human fibroblasts to functional excitatory cortical neurons integrating into human neural networks

**DOI:** 10.1186/s13287-017-0658-3

**Published:** 2017-09-29

**Authors:** Giedre Miskinyte, Karthikeyan Devaraju, Marita Grønning Hansen, Emanuela Monni, Daniel Tornero, Niels Bjarne Woods, Johan Bengzon, Henrik Ahlenius, Olle Lindvall, Zaal Kokaia

**Affiliations:** 1 Laboratory of Stem Cells and Restorative Neurology, University Hospital, Lund, Sweden; 2 Division of Molecular Medicine and Gene Therapy, Lund University, Lund, Sweden; 3 Division of Neurosurgery, Department of Clinical Sciences Lund, University Hospital, Lund, Sweden; 4 Stem Cells, Aging and Neurodegeneration Group, University Hospital, Lund, Sweden; 5 Lund Stem Cell Center, University Hospital BMC B10, Lund University, SE-221 84 Lund, Sweden

**Keywords:** Cortical projection neurons, Direct conversion, Human adult cortical slices, Human fibroblasts

## Abstract

**Background:**

Human fibroblasts can be directly converted to several subtypes of neurons, but cortical projection neurons have not been generated.

**Methods:**

Here we screened for transcription factor combinations that could potentially convert human fibroblasts to functional excitatory cortical neurons. The induced cortical (iCtx) cells were analyzed for cortical neuronal identity using immunocytochemistry, single-cell quantitative polymerase chain reaction (qPCR), electrophysiology, and their ability to integrate into human neural networks in vitro and ex vivo using electrophysiology and rabies virus tracing.

**Results:**

We show that a combination of three transcription factors, BRN2, MYT1L, and FEZF2, have the ability to directly convert human fibroblasts to functional excitatory cortical neurons. The conversion efficiency was increased to about 16% by treatment with small molecules and microRNAs. The iCtx cells exhibited electrophysiological properties of functional neurons, had pyramidal-like cell morphology, and expressed key cortical projection neuronal markers. Single-cell analysis of iCtx cells revealed a complex gene expression profile, a subpopulation of them displaying a molecular signature closely resembling that of human fetal primary cortical neurons. The iCtx cells received synaptic inputs from co-cultured human fetal primary cortical neurons, contained spines, and expressed the postsynaptic excitatory scaffold protein PSD95. When transplanted ex vivo to organotypic cultures of adult human cerebral cortex, the iCtx cells exhibited morphological and electrophysiological properties of mature neurons, integrated structurally into the cortical tissue, and received synaptic inputs from adult human neurons.

**Conclusions:**

Our findings indicate that functional excitatory cortical neurons, generated here for the first time by direct conversion of human somatic cells, have the capacity for synaptic integration into adult human cortex.

**Electronic supplementary material:**

The online version of this article (doi:10.1186/s13287-017-0658-3) contains supplementary material, which is available to authorized users.

## Background

Somatic cells can be directly converted to functional neurons, so-called induced neuronal (iN) cells, by ectopic expression of neural conversion factors [1, 2]. Human-derived iN cells have several attractive features for intracerebral transplantation. Patient-specific neurons can be produced without passing through a pluripotent state, avoiding immune reactions and tumorigenicity as well as the ethical issues associated with the use of human fetal tissue or embryonic stem cells. Neurons generated by direct conversion can also be used for disease modeling [3, 4]. Using forced expression of lineage-specific transcription factors that act during brain development, specific subtypes of neurons, including dopaminergic [5, 6], serotonergic [7, 8], spinal motor [9], cholinergic [10], and striatal medium spiny neurons [11], have been generated directly from human fibroblasts. Recently, small molecules were used not only to improve efficiency of iN cell conversion [12–14] but also to alone convert human [15] and mouse fibroblasts [16] into functional neurons.

Cortical pyramidal neurons have so far not been generated by direct conversion. Given the complexity of the human cortex it is a daunting task to reprogram somatic cells to specific cortical neurons. The cortex has six functionally and morphologically distinct layers with characteristic molecular signatures [17–19]. It is divided into functional areas that form distinct regions during development and are further refined as maturation progresses [19]. Normal cortical development requires precise expression of specific patterns of transcription factors at distinct stages. These transcription factors could possibly be used to convert somatic cells to cortical neurons.

Here we describe the generation of induced cortical (iCtx) cells from human fibroblasts by overexpression of transcription factors involved in the normal development of cortical neurons. The iCtx cells exhibit morphological, molecular, and functional properties of excitatory cortical neurons. Importantly, we demonstrate using co-cultures with human fetal primary cortical neurons (hCtx cells) and ex vivo transplantation to organotypic cultures of human adult cerebral cortex (hACtx) that iCtx cells can integrate into human cortical networks.

## Methods

### Cell culture

#### Derivation of human lung fibroblasts

Human fetal tissue was obtained from Lund and Malmö University Hospitals according to guidelines approved by the Lund-Malmö Ethical Committee. The developmental stage of the fetuses was determined by crown-to-rump length and careful evaluation of external features of the fetuses and internal features of the nervous system. Tissue was microdissected under a stereo-microscope (Leica, Germany) in ice-cold hibernation medium. Human fetal lung fibroblasts (henceforth referred to as HEFL) were isolated from dead aborted human fetuses aged 7–9 week postconception. After removal of the central nervous system and spinal ganglia, the trachea was exposed and resected at the bifurcation. Lungs were then transferred to a clean petri dish, washed several times with cold hibernation medium, and sub-dissected with another set of sterile tools. Great care was taken to remove the residual bronchi. The tissue was transferred several times to a clean petri dish containing sterile cold hibernation medium to remove contaminating cells. The sub-dissected pulmonary tissue was digested with 0.25% trypsin (Sigma-Aldrich) at 37 °C for 10 min and manually triturated to reach a single cell suspension. Cells were plated onto 0.1% gelatin (Sigma) in Dulbecco’s modified Eagle’s medium (DMEM) 4.5 g/L glucose supplemented with 2 mM glutamax and 10% fetal bovine serum (FBS) (all from Life technologies) (herein indicated as human fibroblast expansion medium; hFEM) and were passaged after reaching confluence with 0.25% trypsin. The HEFL cell line was used for neuronal differentiation protocols after passage 3 to avoid contamination from neural tissue and other cell types [2, 6].

#### Induction of cortical neurons

All lentiviral vectors were handled in a class II biosafety laboratory and a multiplicity of infection (MOI) of 2 for each vector was used for all viral transductions. Lentiviral particles with the VSVG capsid were prepared according to a protocol from Dull et al. [20]. The pLD-puro-2A-M2rtTA-TcVA [21] (Addgene plasmid #24592; henceforth referred to as puro-rtTA) was used for selecting cells with puromycin (2 μg/ml) for rtTA expression and used for subsequent reprogramming experiments. The human consensus coding sequences (CCDS) of Fezf2, Ctip2 (also known as BCL11B), NeuroG2 (henceforth referred to as Ngn2), and NeuroD1 (henceforth referred to as Nd1) were synthesized (Genscript, CA) and cloned into the BamHI/PmeI site of pBOB-TRE-WPRE. FUW-TetO-Brn2 and FUW-TetO-Myt1l (Addgene plasmids #27151 and 27152, respectively ([2] #126)) were used along with the above-mentioned vectors in nine different combinations. In addition, cells were transduced with pLemir9-124 (Addgene plasmid #31779) to further enhance the conversion efficiency of the BMF condition. HEFL cells selected with puromycin for rtTA expression were grown to 90% confluency and passaged. Upon passage, cells were transduced with the respective pool of transcription factor, expressing lentiviral vectors and plated to six-well plates with coverslips or T75 flasks. Three days later doxycycline was added to induce expression of the transcription factors and thereby generate cortical neurons from fibroblasts (iCtx cells), and after another 3 days the medium was changed to neuronal induction medium (iCtx medium: Neurobasal, 2% B27 without vitamin A, 0.5 mM glutamine and 100 U/ml penicillin/streptomycin). For small molecule (SM) conditions (single-cell quantitative polymerase chain reaction (qPCR) and co-culture with adult human cortex organotypic slice culture experiments), iCtx medium was supplemented with growth factors at the following concentrations: 10 ng/mL BDNF, 2 ng/mL GDNF, 10 ng/mL NT3 (Peprotech), and 0.5 mM db-cAMP (Sigma). The SMs CHIR99021, SB431542 (Sigma), Noggin (Peprotech), and LDN-193189 (Axon) were added to the media along with growth factors at the same concentrations as previously reported [12]. Cells were cultured in iCtx medium until 25–38 days after adding doxycycline (days postinduction; dpi). Doxycycline was maintained throughout the differentiation protocol and medium was changed every 3 days. Cells were additionally transduced with pHG-hSynI-GFP lentiviral vector (SynI-GFP; kind gift of Dr. Cecilia Lundberg, Lund University) 72 h before electrophysiological analysis in some experiments.

#### Derivation of human fetal primary cortical and striatal cells

Primary cortical (hCtx) and striatal (hStr) cells were derived from cerebral cortex and striatum, respectively, of aborted human fetuses according to guidelines approved by the Lund-Malmö Ethical Committee. The tissue was carefully dissected, minced into small pieces, and then triturated with a pipette tip into a single-cell suspension. The cells were washed with iCtx medium and plated onto poly-d-lysine (PDL; Sigma-Aldrich)/fibronectin (Life Technologies) (both 10 μg/mL)-coated glass coverslips at a density of 20,000 cells per cm^2^ and maintained in iCtx medium until co-culturing. Cells were fixed with 4% paraformaldehyde (PFA) after a week for morphological analysis.

#### Co-culture of iCtx cells and adult human cortex organotypic slice cultures

Adult human cortical tissue (hACtx) was obtained by informed consent from patients undergoing elective surgery for temporal lobe epilepsy (three females and two males, aged 29–52 years) according to guidelines approved by the Lund-Malmö Ethical Committee. The surgically resected tissue was immediately kept in ice-cold dissection medium (L-15 medium, 2% B-27 without vitamin A and penicillin/streptomycin 100 U/mL (Life Technologies)) and transferred to the dissection microscope. The meninges and blood vessels sticking to the pial surface of the brain were carefully removed and the tissue was transferred to the slicing chamber of a Vibratome (Leica VT1200S) filled with ice-cold dissection medium. Slices (250 μm thick) were transferred to 24-well plates containing ice-cold dissection medium with one slice per well. The slices were then transferred to Millicell cell culture inserts (PICM03050, Millipore) in six-well plates containing 1:1 ratio of dissection medium and slice culture medium (Neurobasal medium, 2% B27 without vitamin A, 0.5% N2, 0.5 mM l-glutamine (Life Technologies), 0.1% human albumin (Sigma-Aldrich) with 100 U/mL penicillin/streptomycin) and incubated in 5% CO_2_ at 37 ° C. Twenty-four hours later the medium was completely changed to slice culture medium, which was changed once a week. The hACtx slices were checked for their viability by fixing the slice in 4% PFA and they were then assessed by immunocytochemistry for neuronal (MAP2, FOX3, and SMI 311) and glial (GFAP) markers. The slices were also transduced with SynI-RFP lentiviruses and electrophysiological analysis was performed on RFP^+^ neurons. Non-transduced hACtx slice cultures were used for co-cultures with iCtx cells. The miR124.T-GFP reporter and SynI-RFP transduced 10 dpi HEFL and iCtx cells (BMF combination) were transplanted on top of the slices at 2 weeks after the start of culturing and then co-cultured for another 3 weeks before they were fixed in 4% PFA and assessed by immunocytochemistry. SynI-GFP^+^ iCtx cells, co-cultured with hACtx slices for 3 weeks, were used for electrophysiological recordings. A tracing experiment was performed on hACtx slices with lentivirus FUW-TetO-GFP at days 4 to 6 in culture. hACtx slices were washed with slice culture medium at least twice before co-culture with iCtx cells. We used a polycistronic lentiviral tracing vector [22] and plated newly formed iCtx cells (15 days after doxycycline application) on top of the slices that were kept 2 weeks in culture. At the end of additional 3 weeks in culture, slices were subjected to ΔG-rabies virus and 72 h later fixed and stained.

### RNA extraction and qPCR

Cells grown in six-well plates were washed with ice-cold phosphate-buffered saline (PBS) twice after removal of culture media. RLT buffer (350 μL) was used to lyse the cells in the plate, which were snap frozen on dry ice and stored at –80 °C until extraction of RNA. RNA was extracted with the RNeasy Micro kit (Qiagen) according to the manufacturer’s instructions. RNA extracted from human fetal cortical samples, dissected from aborted human fetuses at 6, 7, 8.5, and 11 weeks postconception, was used as a control. cDNA was synthesized using the iScript Advanced cDNA synthesis kit (Bio-Rad, CA). The cDNA was pre-amplified with TaqMan PreAmp Master Mix (Applied Biosystems, CA) according to the manufacturer’s instructions with TaqMan assays (Applied Biosystems) that would be used for qPCR. The pre-amplified cDNA was diluted 1:20 for qPCR reactions on an iQ5 thermal cycler (Bio-Rad). Ct values of genes were normalized (ΔCt) with geometric mean of Ct values for GAPDH and β-actin. Relative expression (ΔΔCt) to tau (MAPT) within each sample was plotted on Prism (GraphPad).

For single-cell qPCR analysis, candidate genes related to cortical layer or region identity and neuronal function were curated from multiple sources [17–19]. UBC, YWHAZ, and TBP were included as housekeeping genes. COL1A1 was included to identify fibroblasts. Control genes normally expressed in dopaminergic, noradrenergic, serotonergic, and hindbrain neurons were also included. A complete list of TaqMan assays is shown in Additional file 1.

A single-cell suspension was generated using accutase dissociation followed by labeling for NCAM and CD44. Cells were sorted on a FACSAriaI cell sorter (BD Biosciences). Gates were set to include only live (Draq7 (Abcam) negative) cell population. CD44^–^/NCAM^+^/RFP^+^ single cells were sorted into a 96-well PCR plate containing a lysis buffer (0.4% NP40, deoxynucleoside triphosphates, dithiothreitol, and RNase OUT (Invitrogen)) and snap frozen. On thawing, CellsDirect reaction mix containing SSIII/PlatinumTaq (CellsDirect One-Step RT_qPCR kit, no ROX, Invitrogen) and 48 TaqMan assays to a final dilution of 0.05× each were added to the cell lysate for RT-PCR pre-amplification. Zero-, 10- and 50-cell controls were included. RT–PCR pre-amplification cycling conditions were: 50 °C, 60 min; 95 °C, 2 min; 25 × (95 °C, 15 s; 60 °C, 4 min). Individual TaqMan assays were combined with assay loading reagent (Fluidigm) before being pipetted onto a Biomark chip (Fluidigm). The cDNA from the 96-well PCR plate was diluted with water 1 in 5, combined with sample loading reagent (Fluidigm), and loaded onto the same Biomark chip. This was run on a Biomark analyser system (Fluidigm). The arrays were read in a Biomark genetic analysis system (Fluidigm) and the data exported into Microsoft Excel for downstream analysis. The amplification curves were quality controlled and the data filtered according to no-reverse-transcription control reactions and to exclude Ct > 25. Detection thresholds [23] were automatically generated using a baseline linear correction model and a quality threshold of 0.65.

The data were analyzed in Singular (Fluidigm, CA) package in R for hierarchical clustering analysis and single-cell expression visualizer SCExV [24] for principal component analysis (PCA). A well was defined as containing a cell that had been successfully reverse transcribed if there were detectable levels (Ct < 25) of at least two out of three housekeeping genes. Genes detected in no-template controls were excluded from further analysis.

We used three independent viral transduction experiments in order to generate induced neuronal cells and collect samples for bulk qPCR experiments. For single-cell qPCR we sorted cells from two independent viral transduction experiments and used four chips of 48 × 48 (Fluidigm) to run the analysis.

### Immunocytochemistry

Cultures were washed with PBS and fixed in 4% PFA for 20 min. The coverslips were washed three times with PBS, permeabilized with 0.025% TritonX-100 in PBS for 10 min, and then blocked with 5% normal donkey serum for 45 min at room temperature. The primary antibodies (mouse anti-MAP2a/b (MAP2; 1:500, Sigma-Aldrich), chicken anti-MAP2 (1:5000, Abcam, UK), mouse anti-βIII-tubulin (1:500, Covance, NJ), rabbit anti-βIII-tubulin (1:2000, Covance, NJ), mouse anti-Satb2 (1:20, Abcam), chicken anti-GFP (1:3000, Abcam), rabbit anti-RFP (1:1000, Abcam), mouse anti-PSD95 (1:100, Abcam), chicken anti-Tbr1 (1:1000, Millipore), and rat anti-Ctip2 (1:500, Abcam)) diluted in blocking solution were applied overnight at 4 °C. For Satb2 and Tbr1 immunostaining, antigen retrieval with 10 mM citrate buffer (pH 6.0) was performed. After three rinses with PBS, Alexa488, Cy3, and Cy5 conjugated donkey or goat secondary antibodies (1:500, Jackson Immunoresearch, PA) against the respective primary antibodies diluted in blocking solution were applied for 1.5 h. Streptavidin-conjugated Alexa647 was used for labeling cells filled with biocytin from electrophysiology experiments. Cell nuclei were counterstained with Hoechst33342 and mounted with PVA-Dabco. For the hACtx slice cultures and co-cultures of iCtx cells with hACtx slice cultures, slices were fixed in 4% PFA overnight and then rinsed three times in PBS for 15 min. The slices were incubated for 48 h with the primary antibodies (chicken anti-MAP2 (1:1000, Abcam), mouse anti-SMI 311 (pan-neuronal neurofilament; 1:400, Covance), mouse anti-GFAP (1:400, Sigma), chicken anti-GFP (1:1000, Abcam), and rabbit anti-RFP (1:500, Abcam)) in 0.5% TritonX-100 in PBS and 5% normal donkey and goat sera at 4 °C. The slices were washed again in 0.5% Triton X-100 in PBS twice and incubated in Alexa488 and Cy3-conjugated respective secondary antibodies for 48 h at 4 °C. The slices were washed three times in PBS and incubated in Hoechst 33342 for 15 min at room temperature. Slices were mounted on glass slides with PVA-Dabco after rinsing with deionised water.

### Microscopy

Images were obtained on BX61 epifluorescence (Olympus, Japan) and LSM780 confocal (Zeiss, Germany) microscopes. Co-cultures of iCtx cells with cortical slice cultures were imaged with a LSM780 confocal microscope (Zeiss). Optical sections were made and *z*-stacks were rendered for three-dimensional reconstruction using Imaris (Bitplane, Switzerland).

#### Conversion efficiency

In vitro quantification was performed in at least 20 regions of interest from three different coverslips per condition in an epifluorescence microscope. The total number of MAP2/βIII Tubulin^+^ cells with neuronal morphology was quantified at 25–30 dpi. Conversion efficiency was calculated as previously described [2, 25]. Briefly, the average number of neuronal cells present in 20 randomly selected 20× visual fields was estimated. The area of the 20× visual field was then used to determine MAP2^+^/βIII Tubulin^+^ cell density in the entire dish. This number was divided by a number of plated cells during transduction to obtain the percentage of the starting population of cells that acquired neuron-like characteristics.

#### Immunoreactivity assay

Assessment of MAP2/βIII Tubulin immunoreactivity was performed using cellSens Dimension (Olympus, Japan) imaging software to determine the neurite density. Twenty images of randomly chosen regions in three representative coverslips were acquired. Using a 10× objective, 15 fields were chosen randomly in three coverslips for each condition. In each coverslip, areas of MAP2/βIII Tubulin immunoreactivity were identified using defined representative ranges of the threshold for the specific signal. Using these defined parameters, the images of each area were analyzed by software, which calculated the total area covered by the specific immunopositive signal (*X* in μm^2^). The number of MAP2/βIII Tubulin cells (*Y*) that had Hoechst 33342-positive nuclei in each field was counted. This number was then used to calculate the immunoreactivity area per cell (X/Y μm^2^/cell) to obtain a measure of neurite density.

#### Pyramidal morphology index

The pyramidal morphology index (PMI) was defined as the ratio between the width of the largest process and the total number of processes crossing a sampling circle [26] in CellSens imaging software (Olympus, Japan). To determine the PMI, at least 25 BMF-induced iCtx cells and hCtx neurons were randomly chosen. The number of processes crossing the sampling circle was counted and the width of the widest neurite was measured. The index was calculated for multipolar cells that had more than two processes.

#### Cell soma size

The cell soma size was measured from 25 cells randomly chosen from three coverslips of BMF-induced iCtx cells and hCtx neurons in CellSens imaging software. Images were obtained using a 20× objective and the outline of the cell body was delineated manually. The area corresponding to the cell soma was calculated by the software and used for analyzing the average cell soma size.

#### Neuronal morphology tracing

Neuronal morphology was assessed for three representative BMF-induced iCtx cells and hCtx and hStr neurons using simple neurite tracer plugin in Fiji [27]. The outlined areas were filled out and then converted to 8-bit monochrome image in Fiji.

### Electrophysiology

Whole-cell patch-clamp recordings were performed with a HEKA double patch clamp EPC10 amplifier using PatchMaster for data acquisition. Cells were grown on coverslips and transferred to the recording chamber. The coverslip was constantly perfused (1 ml/min) with carbogenated artificial cerebrospinal fluid (aCSF, in mM: 119 NaCl, 2.5 KCl, 1.3 MgSO_4_, 2.5 CaCl_2_, 26 NaHCO_3_, 1.25 NaH_2_PO_4_, and 25 glucose (11 glucose for recordings in hACtx slices), pH 7.2–7.4, 295–300 mOsm) at 34 °C. Recording pipettes were filled with intracellular solution containing (in mM): 122.5 KGlu, 12.5 KCl, 10.0 HEPES, 8.0 NaCl, 2.0 MgATP, and 0.3 Na_2_GTP for recordings of intrinsic properties; 135 CsGlu, 10 HEPES, 10 NaCl, 1 MgCl_2_, 2 MgATP, and 0.4 Na_2_GTP for recordings of evoked excitatory postsynaptic currents (EPSCs); and 135 CsCl, 10 HEPES, 10 NaCl, 2 MgATP, and 0.3 NaGTP for recordings of evoked inhibitory postsynaptic currents (IPSCs) and spontaneous postsynaptic currents (sPSCs). Intracellular solutions had pH of 7.2–7.4, osmolarity of 285–295 mOsm, and resistance of 2.5–9.5 MΩ. Biocytin (2–4 mg/ml) was added to the internal solution prior to recording for post hoc identification of the recorded cell. QX314 (5 mM) was added to the internal solution before recording of sPSCs. Voltage values were not corrected for the liquid junction potential, which was 13.82 mV, 15.55 mV, and 5.10 mV for KGlu-, CsGlu- and CsCl-based internal solutions, respectively. Only cells with a series resistance below 30 MΩ were included in the analysis (BMF-induced cells: Rseries = 15.0 ± 1.2 MΩ, *n* = 29).

Voltage- and current-clamp recordings were used for electrophysiological characterization. Sodium and potassium currents were evoked by a series of 200-ms long voltage steps (from –70 mV to +40 mV in 10-mV steps) and their sensitivity to 1 μM tetrodotoxin (TTX) and 10 mM tetraethylammonium (TEA), respectively, was determined. A series of current steps (0– 200, 400, or 1000 pA in 10, 20, or 50 pA steps) lasting 500 ms were performed from a membrane potential of ~ –70 mV (current was injected when needed to keep the membrane potential ~ –70 mV) to determine the ability of the cell to generate action potentials (APs). EPSCs and IPSCs were evoked by puff application (0.5–0.75 bar) of 100 mM glutamate or 100 mM GABA lasting 0.5–1 s using a pneumatic drug ejection system (PDES-02DE-2). sPSCs were recorded from a holding potential of –70 mV. AMPA and NMDA receptors were blocked by 5 μM 2,3-dioxo-6-nitro-1,2,3,4-tetrahydrobenzo[*f*]quinoxaline-7-sulfonamide (NBQX) and 50 μM d-(–)-2-amino-5-phosphonopentanoic acid (D-APV), respectively. GABA receptors were blocked by 0.1 mM picrotoxin (Ptx). Data were analyzed offline with Fit-Master, IgorPro 6.3, and NeuroMatic v2.8b.

### Statistics

Statistical analysis was performed using one-way analysis of variance (ANOVA) followed by Tukey’s multiple comparisons test or uncorrected Fisher’s LSD, Kruskal-Wallis test followed by Dunn’s multiple comparison test, or one-sample *t* test in Prism 6 software (GraphPad). Significance was set at *p* < 0.05. Data are shown as mean ± SEM calculated by subtracting Ct of a sample with the mean Ct value of all samples for a given gene and dividing it by the corresponding standard deviation.

## Results

### Combinations of transcription factors convert human fibroblasts to functional neurons with cortical-like phenotype

**Fig. 1:**
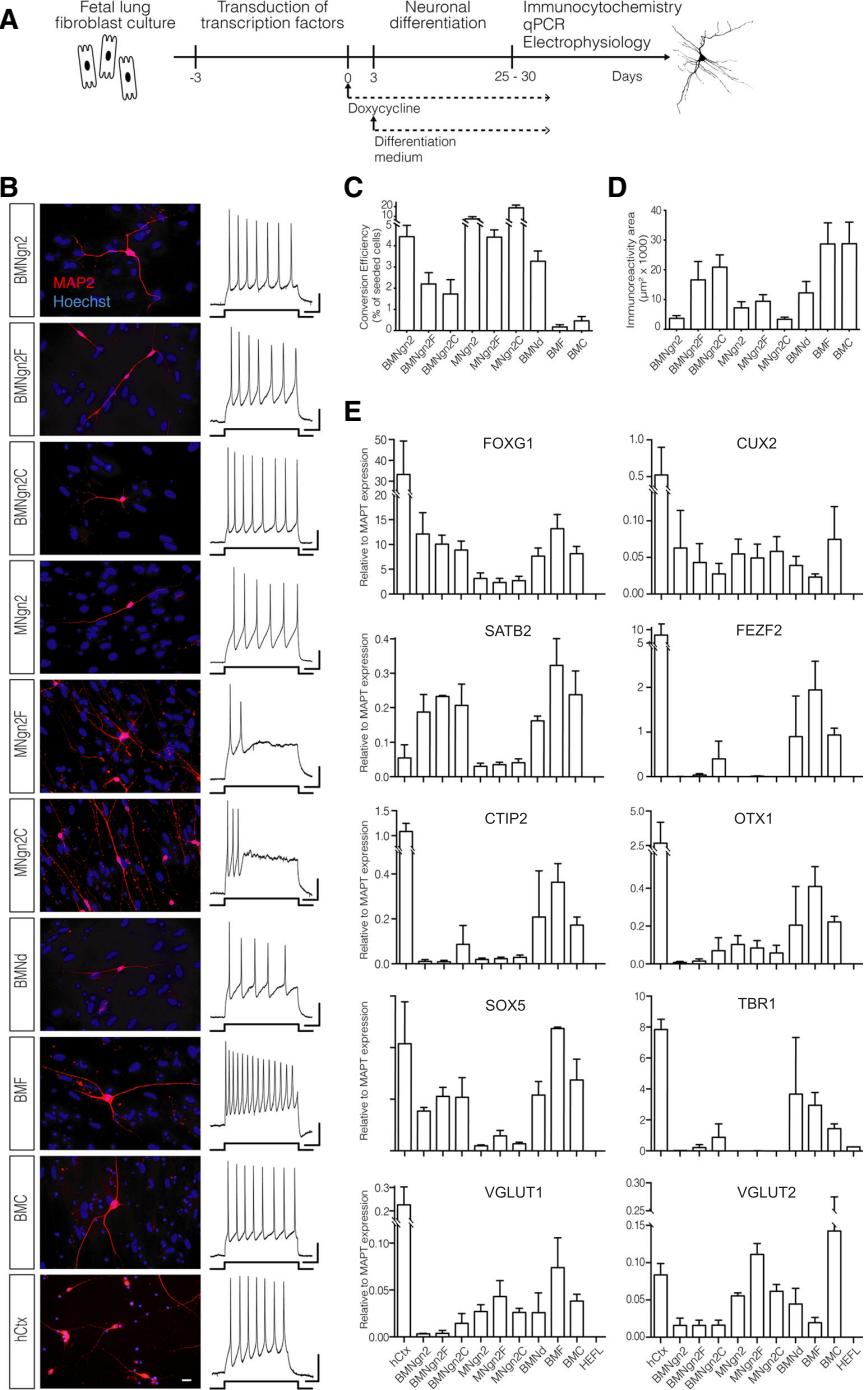
Human neuronal like cells with cortical characteristics are induced by different transcription factor combinations. **a** Experimental design. **b** Nine different combinations of transcription factors convert human fibroblasts to MAP2^+^ neurons (*scale bar* = 20 μm), which generate action potentials upon 500 ms of 40, 80, 40, 30, 20, 40, 40, 200, and 140 pA current injections for BMNgn2, BMNgn2F, BMNgn2C, MNgn2, MNgn2F, MNgn2C, BMNd, BMF, and BMC, respectively. Human fetal primary cortical (*hCtx*) neurons for comparison (20 pA current injection) (horizontal bar: 0.1 s; vertical bar: 20 mV). **c**–**e** Neuronal conversion efficiency (*n* = 5) (**c**), neurite density as measured by immunoreactivity area (*n* = 3) (**d**), and expression of cortical markers (*n* = 3) (**e**), as revealed by quantitative polymerase chain reaction (*qPCR*), induced by different transcription factor combinations. Data re shown as mean ± SEM; *n* corresponds to a number of independent differentiation experiments

We designed an experimental set up (Fig. 1a) with the aim of identifying transcription factors that would reprogram human fibroblasts to glutamatergic cortical neurons. We composed nine combinations (Fig. 1b) of cortical layer- and development-specific transcription factors including Brn2 (B) and/or Myt11 (M) along with Fezf2 (F), Ctip2 (C), NeuroG2 (Ngn2), and NeuroD1 (Nd). Doxycycline-inducible (tet-on) lentiviral vectors encoding the different combinations were transduced to puro-rtTA-expressing human fetal lung fibroblasts (HEFL) (Fig. 1a). At 25–30 days postinduction (dpi), cells were stained for the neuron-specific protein MAP2.

All combinations of transcription factors gave rise to MAP2^+^ cells with neuronal morphology (Fig. 1b). Some transcription factor combinations showed greater conversion efficiency, but the generated MAP2^+^ cells were bipolar with small soma. The BMF and BMC combinations exhibited low conversion efficiency, while the cells were multipolar with pyramidal morphology and extensive neurite density (Fig. 1b–d). Whole-cell patch-clamp recordings revealed that many MAP2^+^ cells produced one or more APs (Fig. 1b). The input resistance and membrane capacitance varied between some of the transcription factor combinations, but they all had average resting membrane potential, input resistance, and membrane capacitance similar to those of primary human fetal cortical neurons (hCtx) (Table 1). The majority (62–89%) of MAP2^+^ cells induced in the presence of BRN2 generated multiple APs, whereas only 40–44% of cells converted without BRN2 were able to generate multiple APs upon current injection (Table 1). We observed no difference in the maximum number of APs generated by MAP2^+^ cells and hCtx cells (Table 1). Taken together, our findings indicate that all tested transcription factor combinations produced functional iN cells.

**Table 1:** Electrophysiological properties and AP characteristics of induced neuronal cells

	BMNd	BMF	BMC	BMNgn2	BMNgn2F	BMNgn2C	MNgn2	MNgn2F	MNgn2C	hCtx
Days in vitro^a^	25–31	26–37	26–38	25–32	27–33	28–34	27–32	27–33	29–34	20–23
Number of coverslips	4	4	5	10	3	3	3	4	4	4
Number of cells	10	11	11	26	9	13	7	9	5	8
V_rest_ (mV)	–45.3 ± 2.2	–44.1 ± 3.74	–48.3 ± 5.6	–42.6 ± 1.9	–38.6 ± 3.9	–47.4 ± 3.3	–36.9 ± 3.0	–33.3 ± 2.7	–35.3 ± 5.0	–43.9 ± 2.6
R_input_ (MΩ)	798 ± 156*	229 ± 29	507 ± 107	686 ± 66*	441 ± 96	623 ± 127	908 ± 120*	800 ± 139*	781 ± 224	644 ± 70
Capacitance (pF)	9.5 ± 1.7	42.0 ± 15.8	25.0 ± 10.7	6.8 ± 1.4*^$^	35.1 ± 10.2	26.7 ± 10.7	3.0 ± 0.6*^$^	5.3 ± 2.4*^$^	3.7 ± 0.8*	6.8 ± 2.1
Cells with more than 1 AP, % (*n*/*N*)	80 (8/10)	82 (9/11)	73 (8/11)	65 (17/26)	89 (8/9)	62 (8/13)	43 (3/7)	44 (4/9)	40 (2/5)	75 (6/8)
Number of APs	5.8 ± 1.0*	16.0 ± 3.2	10.6 ± 3.2	6.0 ± 1.2*	5.7 ± 1.5*	7.4 ± 2.7	4.0 ± 1.8*	1.8 ± 0.4*	2.6 ± 1.4*	6.1 ± 1.4
Threshold	–21.4 ± 1.8	–20.5 ± 1.7	–22.0 ± 1.6	–21.2 ± 1.1	–21.6 ± 1.8	–20.6 ± 1.3	–22.6 ± 2.7	–20.7 ± 1.7	–18.8 ± 1.3	–24.5 ± 1.6
Amplitude	39.9 ± 3.4*^£^	58.5 ± 4.7	60.4 ± 5.4	48.8 ± 2.7^£^	50.8 ± 5.6	48.6 ± 5.4	44.7 ± 4.1^£^	39.9 ± 4.4*^£^	37.3 ± 5.2*^£^	26.9 ± 7.1
Rise time	1.7 ± 0.3	1.4 ± 0.3	1.6 ± 0.4	1.4 ± 0.1	1.4 ± 0.1	1.4 ± 0.1	1.7 ± 0.2	2.1 ± 0.3	1.6 ± 0.2	1.6 ± 0.2
Half amplitude width	2.3 ± 0.3	2.1 ± 0.4	3.1 ± 0.7	1.7 ± 0.1	1.9 ± 0.3	1.6 ± 0.1	2.0 ± 0.4	2.1 ± 0.2	2.0 ± 0.3	2.2 ± 0.3
After hyperpolarization peak	21.3 ± 3.1	23.5 ± 1.9	18.2 ± 2.8	17.9 ± 1.1	18.2 ± 2.0	19.0 ± 2.5	16.2 ± 3.9	15.7 ± 2.4	18.4 ± 3.8	9.0 ± 2.7

^a^ Day 0 corresponds to the first day after induction

Resting membrane potential (V_rest_), one-way ANOVA *p* = 0.0554

Input resistance (R_input_), Kruskal-Wallis test *p* = 0.0016, Dunn’s multiple comparison test *p* < 0.05

Capacitance, Kruskal-Wallis test *p* < 0.0001, Dunn’s multiple comparison test *p* < 0.05.

Number of action potentials (APs), one-way ANOVA *p* = 0.0011, Turkey’s multiple comparisons test *p* < 0.05. F

AP threshold, Kruskal-Wallis test. *p* = 0.8026

AP amplitude, one-way ANOVA *p* = 0.0154, uncorrected Fisher’s LSD *p* < 0.05.

AP rise time (0–100%), Kruskal-Wallis test *p* = 0.1343

Half AP amplitude width, Kruskal-Wallis test *p* = 0.3701

After hyperpolarization peak, Kruskal-Wallis test *p* = 0.5411

* Statistical difference from B; ^$^ statistical difference from BMNgn2F; ^£^ statistical significance from BMC

The generated iN cells were analyzed for their expression of cortical markers using qPCR on cDNA from unpurified iN and hCtx cultures (Fig. 1e). All combinations of transcription factors yielded cells expressing the forebrain marker FoxG1. The BMNd-, BMF-, and BMC-induced cells predominantly expressed the deep-layer projection neuronal markers SOX5, FEZF2, CTIP2, OTX1, and TBR1, and the callosal projection neuronal marker SATB2. We found the BMF-induced iN cells (henceforth referred to as iCtx) to be particularly interesting. These cells expressed not only projection neuronal markers, especially SOX5, CTIP2, and OTX1, at comparatively higher levels and exhibited a glutamatergic phenotype (VGLUT1 expression), but also had electrophysiological properties similar to those of hCtx cells. Therefore, we chose BMF as the most promising transcription factor combination for generating iCtx cells.

**Fig. 2:**
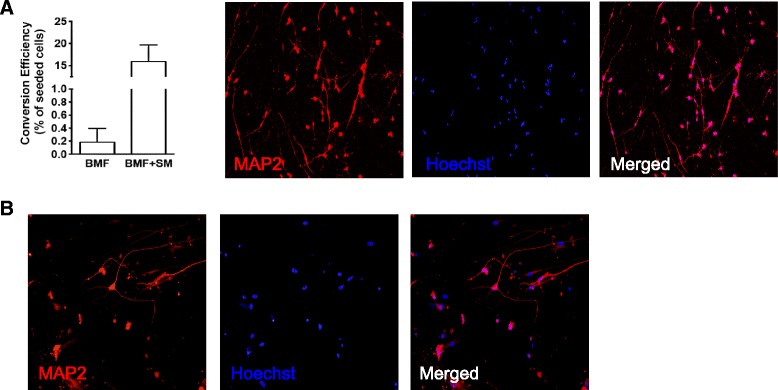
Small molecules and microRNAs improve the conversion efficiency and enable generation of iCtx from adult human skin fibroblasts. **a** Conversion efficiency of iCtx cells generated from HEFL cells with and without small molecule (*SM*) treatment (*n* = 3) (*left*). MAP2-positive cells generated by BMF along with small molecules and microRNAs (*right*). **b** MAP2-positive cells generated by BMF and small molecules from adult human skin fibroblast. *Scale bar* = 20 μm

We then wanted to improve the conversion efficiency for generating iCtx cells from human fibroblasts by BMF combination. The addition of SMAD inhibitors and canonical WNT signaling activators in the form of small molecules [12] or microRNAs [23] has previously been shown to increase the conversion efficiency of iN cells. We tested small molecule treatment (CHIR99021, SB431542, Noggin, and LDN193189) alone or with microRNAs miR-9/9* and miR-124 (miR-9/9*-124) when converting fibroblasts using BMF. These treatments gave rise to a substantial increase in conversion efficiency up to 15.9 ± 3.7% (Fig. 2a). We also found that, using small molecules and microRNAs, it was possible to directly convert adult human skin fibroblasts to iN cells by BMF combination (Fig. 2b).

### A subpopulation of human iCtx cells shares molecular phenotype with human fetal primary cortical neurons

We evaluated in detail the iCtx cells generated by BMF in combination with small molecules and microRNAs, and explored their molecular signature and heterogeneity. For this, we quantitatively analyzed expression of 48 genes (see Additional file 1) involved in cortical development and reflective of subtype and area identity at the single-cell level. We used NCAM and miR-9/9*-124-RFP as positive markers to sort iCtx cells and CD44 [28] to exclude non-reprogrammed fibroblasts from the downstream analysis (CD44^–^/NCAM^+^/RFP^+^). We also included human fetal fibroblasts (HEFL) and hCtx cells from 11-week-old human fetal brain in the single-cell analysis (sorted on CD44^+^ and NCAM^+^, respectively).

**Fig. 3:**
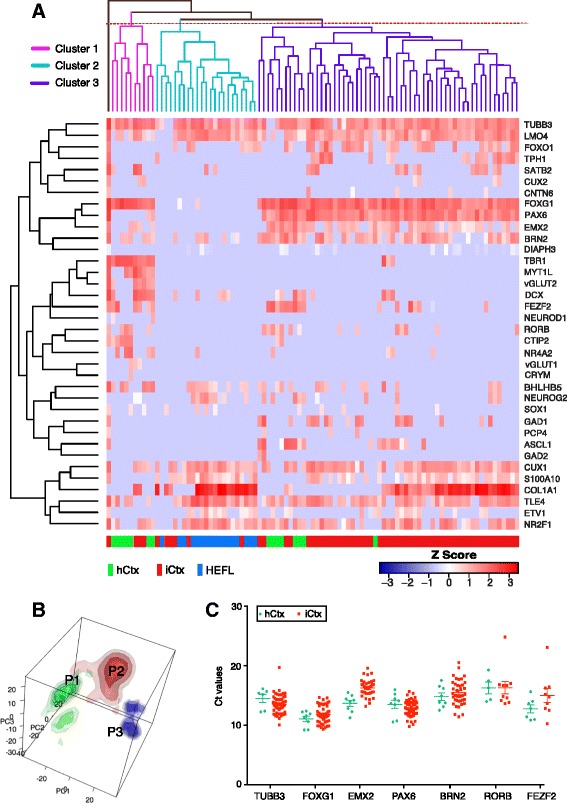
Single-cell quantitative RT-PCR analysis. **a** Hierarchical clustering analysis of the relative mRNA levels (expressed as *z*-Score) in FACS-sorted induced cortical (*iCtx*) cells after 4 weeks of induction. The *red* color indicates higher expression and *white* indicates lower expression of a given gene for the various samples. All cells group into three main clusters. iCtx and human fetal primary cortical (*hCtx*) cells spread in two clusters (1 and 3) while a third one mainly contains human fetal lung fibroblast (*HEFL*) cells (cluster 2). **b** Three-dimensional principal component analysis (PCA) density plot demonstrating subpopulations of iCtx (*red*), hCtx (*green*), and HEFL (*blue*) cells. A subpopulation of iCtx cells (P1) is proximal to hCtx cells, indicating a high degree of similarity in overall gene expression patterns. HEFL and hCtx cells are the most distant populations. The majority of iCtx cells group away from HEFL cells (P2); only a small fraction of iCtx cells shares gene expression profile with HEFL cells (P3). **c** Relative gene expression levels in iCtx (*red dots*) and hCtx (*green dots*) cells for selected neuronal and cortical genes represented as Ct values. Each dot represents a single cell (cluster 3)

Analysis of 61 iCtx cells revealed a complex pattern of gene expression. Using unsupervised hierarchical clustering analysis we identified three main clusters. Two of these contained both iCtx and hCtx cells (clusters 1 and 3). The third cluster (cluster 2) contained iCtx and HEFL cells but not hCtx cells (Fig. 3a). Next, we subjected the single-cell data to PCA (Fig. 3b). In PCA we identified three main populations of iCtx cells. One subpopulation representing 13.1% of iCtx cells exhibited a gene expression profile closely resembling that of hCtx cells (Fig. 3b, P1). Importantly, several cortical genes such as EMX2 and PAX6 (cortical neural progenitor markers), FOXG1 (telencephalon marker), and RORB (layer 4 marker) were expressed in iCtx cells at levels comparable to those in hCtx cells (Fig. 3c). These iCtx cells did not express markers for the dopaminergic (NURR1) subtype of neurons (Fig. 3a). About 10% of iCtx cells grouped with HEFL cells (Fig. 3b, P3), indicating that they had failed to reprogram. The remaining iCtx cells shared features of both HEFL and hCtx cells (Fig. 3b, P2), which suggests partial reprogramming.

### Human iCtx cells mature to functional neurons resembling human fetal primary cortical neurons and form synaptic contacts

**Fig. 4:**
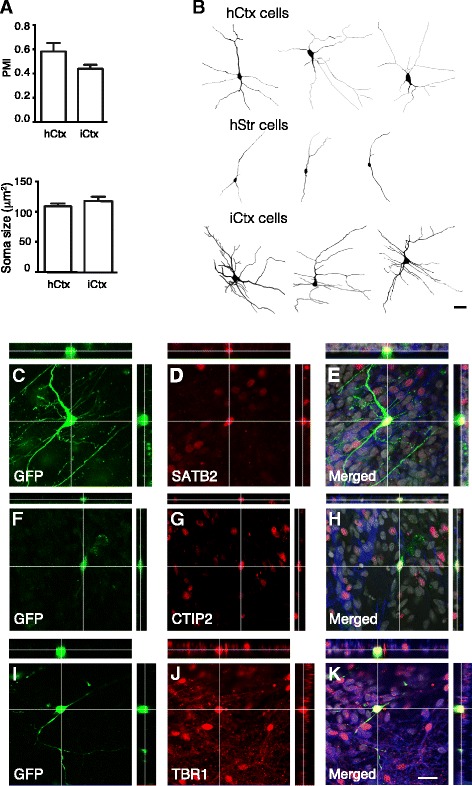
Human cortical pyramidal-shaped neurons are induced by BMF combination. **a** Pyramidal morphology index (*PMI*) and soma size of induced cortical (*iCtx*) cells compared to human fetal primary cortical (*hCtx*) cells. Data are shown as mean ± SEM (*n* = 3). **b** Tracing showing the pyramidal shape and complex morphology of the iCtx cells which are different from the human striatal (*hStr*) cells but resembling those of the hCtx cells. **c–k** Orthogonal projections of confocal images showing expression of the deep-layer cortical neuronal markers SATB2 (**c–e**), CTIP2 (**f-h**). and TBR1 (**i–k**) by iCtx cells, labeled with green fluorescent protein (*GFP*) under human Synapsin-I promoter. *Scale bar* = 20 μm

To promote maturation of BMF-induced iCtx cells, we co-cultured them with hCtx cells for 3 weeks. The shape and size of the iCtx cells were compared with those of hCtx cells. We used the pyramidal morphology index (PMI), an unbiased numerical characterization of dendritic morphology that takes into account the number of neurites emerging from the cell body and the thickness of the apical dendrite [26]. No differences were detected between iCtx and hCtx cells using this index (Fig. 4a). Besides a characteristic pyramidal shape, cortical projection neurons are known to have larger soma size compared to other neurons. Morphometric measurements revealed that the iCtx and hCtx cells had similar soma size (Fig. 4a). When compared with human fetal primary striatal neurons (hStr cells), the iCtx cells exhibited multipolar pyramidal morphologies and rich arborizations similar to hCtx cells, whereas hStr cells were mostly bipolar without extensive arborizations (Fig. 4b). We found by immunocytochemistry that some iCtx cells expressed the callosal projection neuronal marker SATB2 (Fig. 4c–e) and the deep-layer projection neuronal markers CTIP2 (Fig. 4f–h) and Tbr1 (Fig. 4i–k). Taken together, our data indicate that iCtx cells adopt a phenotype resembling that of developing human cortical projection neurons.

**Fig. 5:**
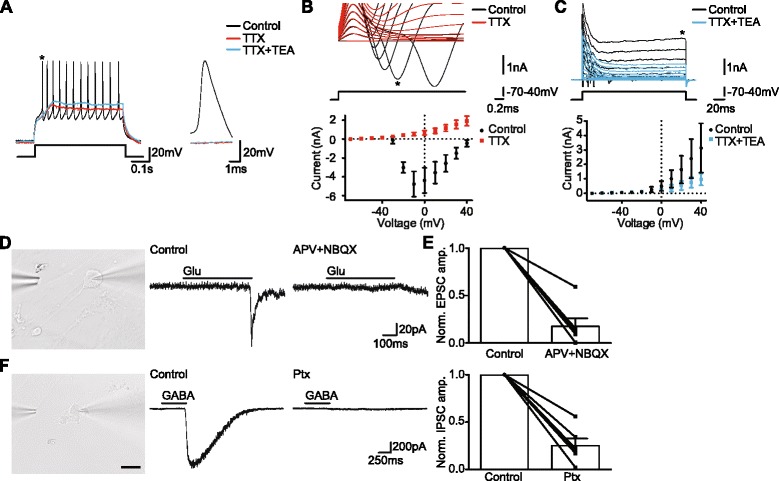
Human BMF-derived iCtx cells are mature neurons and have functional GABA and glutamate receptors. **a** Voltage traces illustrating the generation of APs (*black trace*). APs are blocked by tetrodotoxin (*TTX*) (*red trace*) or TTX and tetraethylammonium (*TEA*) (*blue trace*). * The expanded AP. **B,c** (*top panels*) Current traces of the fast inward current peak (Na current, **b**) and the sustained outward current (K current, **c**) activated by step depolarizations in the absence (*black*) and presence of TTX (*red*, **b**) or TTX and TEA (*blue*, **c**). * Fast inward (**b**) or sustained outward (**c**) current. (*Bottom panels*) Current/voltage (I/V) plot of the fast inward (**b**) and the sustained outward (**c**) current in the absence (*black*) and presence of TTX (*red*, **b**) or TTX and TEA (*blue*, **c**). TTX blocks the fast inward current (*n* = 6, **b**) and TEA inhibits the sustained outward current (*n* = 4, **c**). **d,f** (*left panels*) Pictures of the patched cell and the position of the puff pipette. *Scale bar* = 50 μm. (*Middle panels*) Current trace from the cell shown to the left. Puff application of glutamate (**d**) or GABA (**f**) induces an excitatory postsynaptic current (*EPSC*) (**d**) or inhibitory postsynaptic current (*IPSC*) (**f**) under control conditions (*left traces*). Bath application of D-APV and NBQX blocks the glutamate-induced EPSC (**d**) and picrotoxin (*Ptx*) blocks the GABA-induced IPSC **(f**) (*right traces*). (*Right panels*) Bar chart summarizing data across experiments (*n* = 6, one sample *t* test, *p* = 0.0002 (**d**) and *p* = 0.0002 (**f**)). Presence of D-APV and NBQX (**d**) or Ptx (**f**) inhibits EPSC or IPSC amplitude, respectively

Whole-cell patch-clamp recordings showed that APs in the BMF-induced iCtx cells were blocked by TTX (Fig. 5a), and that these cells expressed both TTX-sensitive fast inward sodium current (Fig. 5b) and TEA-sensitive sustained outward potassium current (Fig. 5c). We tested whether the iCtx cells expressed functional glutamate and GABA receptors. The iCtx cells were labeled with SynI-GFP lentiviral vector to ensure that patched cells were mature neurons (Fig. 5e). Puff application of glutamate evoked an excitatory postsynaptic current (EPSC) in 88% of iCtx cells. The EPSCs were inhibited by D-APV and NBQX (Fig. 5d), illustrating that they were generated by activation of NMDA or AMPA receptors, respectively. Moreover, puff application of GABA evoked an inhibitory postsynaptic current (IPSC) in 75% of iCtx cells. The evoked IPSCs were inhibited by the GABA_*A*_ receptor antagonist picrotoxin (Ptx) (Fig. 5f).

**Fig. 6:**
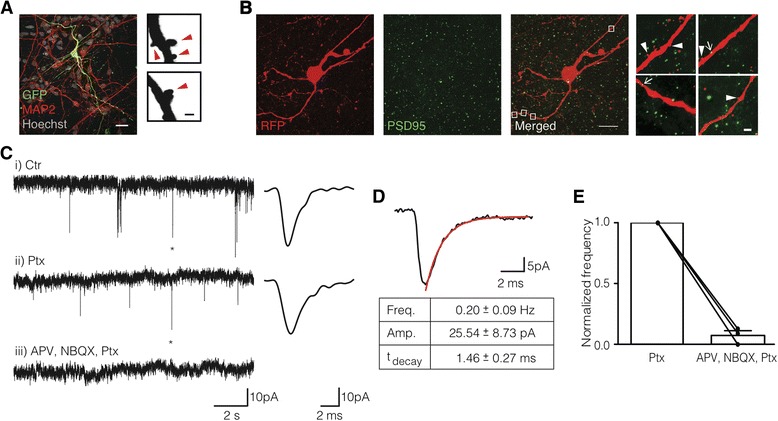
Human BMF-derived iCtx cells form synaptic contacts with human fetal cortical neurons. **a** iCtx cells (GFP^+^ and MAP2^+^), co-cultured with hCtx cells (MAP2^+^), have spines in their neurites. Enlarged images of the spines (*red arrowheads*). *Scale bars* = 20 μm and 1 μm for the iCtx cell and the enlargements, respectively. **b** SynI-RFP^+^ iCtx cells express PSD95 in their neurites (*arrows*). *Arrowheads* indicate spines. *White boxes* indicate enlarged neurites. *Scale bars* = 20 μm and 1 μm for enlargements. **c** Whole-cell patch clamp recordings from iCtx cells co-cultured with hCtx cells. Current traces show generation of spontaneous PSCs (sPSCs) (i). Isolated glutamatergic sPSCs were recorded in the presence of picrotoxin (*Ptx*) (ii), and are blocked by D-APV and NBQX (iii). * Expanded fast decaying sPSC shown on the right. **d** Averaged current trace of fast decaying sPSCs observed under control conditions (*Ctr*) (i). Time constant (*t*_*decay*_) was determined by fitting an exponential (*red*) curve to the decay. Table summarizes data across experiments (*n* = 5 cells). **e** Bar chart summarizing data across experiments (*n* = 3, one sample *t* test, p = 0.0017). D-APV and NBQX blocked glutamatergic sPSCs in all cells tested. Data are shown as mean ± SEM

We wanted to know if iCtx cells could form synaptic connections. Stubby and filopodia types of spines (Fig. 6a) were found on neurites of SynI-GFP^+^ iCtx cells co-cultured with hCtx cells. To check if these spines can receive excitatory synaptic inputs, we immunostained for the postsynaptic excitatory scaffolding protein (PSD-95), which is concentrated at glutamatergic synapses [29]. Many iCtx cells had PSD-95 puncta in neurites in close proximity to spines (Fig. 6b).

To test if the synapses were functional, we recorded from SynI-GFP^+^ iCtx cells co-cultured with hCtx cells. Fast decaying, glutamatergic-like spontaneous postsynaptic currents (sPSCs) were observed in 38% of patched cells (Fig. 6c and d). Isolated glutamatergic sPSCs, recorded in the presence of Ptx, were abolished in the presence of Ptx, D-APV, and NBQX (Fig. 6c and e). These recordings provide evidence that the iCtx cells can develop to functionally mature neurons that establish afferent synaptic connections with hCtx cells.

### Transplanted human iCtx cells integrate into organotypic cultures of adult human cortex and receive synaptic inputs from host cortical neurons

**Fig. 7:**
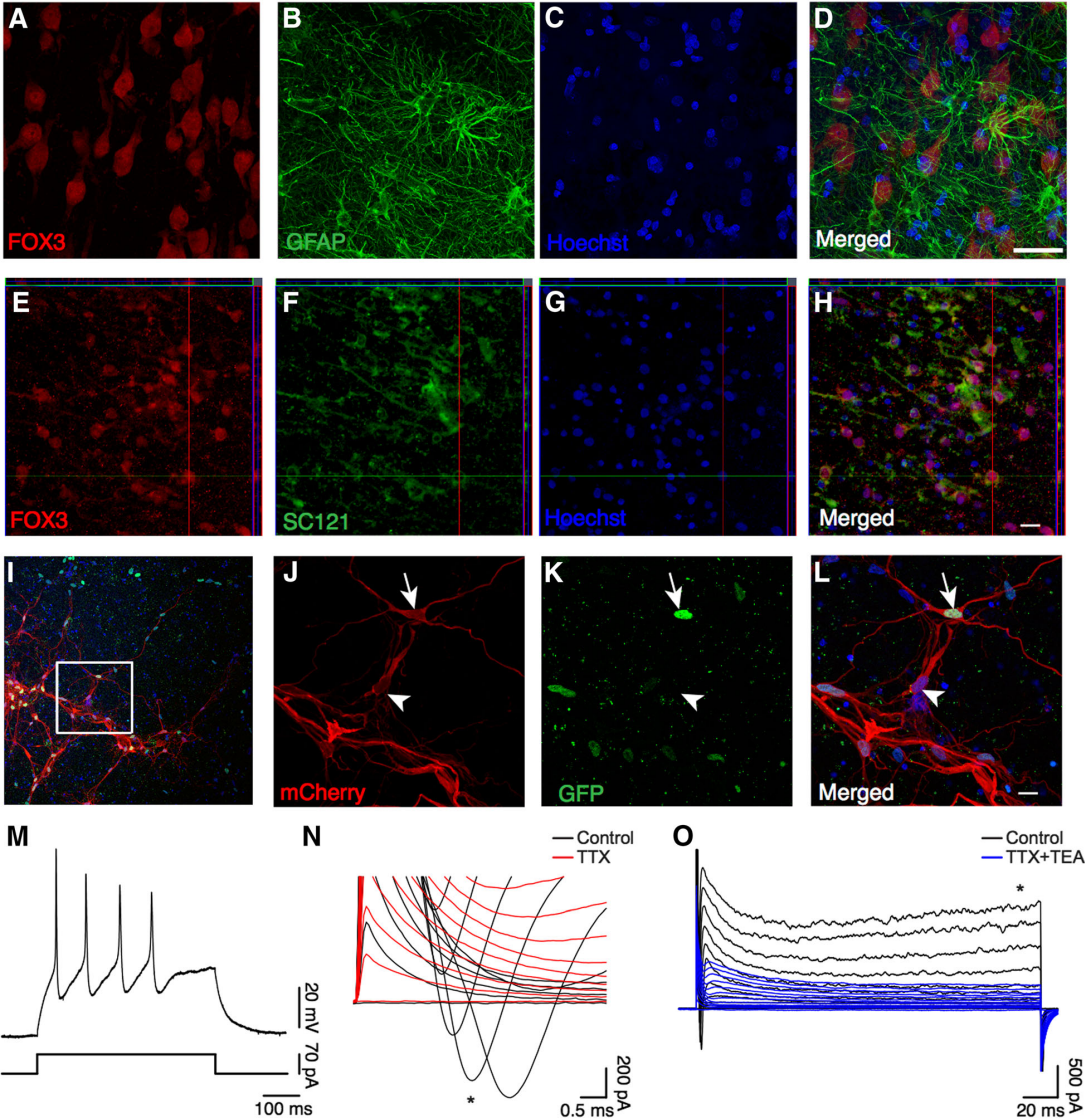
Human adult cortex-derived slice cultures (hACtx) are viable and contain functional neurons and astrocytes up to 6 weeks. **a–d** Confocal images of hACtx slices prior to culture showing expression of Fox3 (**a**), GFAP (**b**), and Hoechst (**c**) by hACtx cells. *Scale bar* = 40 μm. **e–h** Orthogonal projections of confocal images showing expression of FOX3, SC121, and Hoechst by hACtx slices after 6 weeks in culture. *Scale bar* = 20 μm. **i–l** Synaptic connectivity between host neurons in non-grafted hACtx slice cultures. **i** Confocal image of hACtx organotypic slice showing an overview of hACtx cell labeling with TVA-GFP and subsequent tracing with ΔG-rabies virus. Labeled neurons were mCherry^+^/GFP_nuc_^+^ after tracing with ΔG-rabies virus **(l,** *arrow* **)**. Traced neurons were mCherry^+^/GFP_nuc_^–^ (**l**, *arrowhead*), indicating occurence of synaptic connections between adult neurons in the organotypic slice cultures. **m–o** Whole-cell patch-clamp recordings from SynI-RFP^+^ hACtx neurons. **m** Voltage traces illustrating the generation of APs during a 900 pA current step lasting 500 ms. **n,o** Current traces of the fast inward current peak (**n**) and the sustained outward current (**o**) activated by step depolarizations from a holding potential of –70 mV in the absence (*black*) and presence of 1 μM tetrodotoxin (*TTX*) (*red*, **n)** or 1 μM TTX and 10 mM tetraethylammonium (*TEA*) (*blue*, **o)**. * Inward current peak (**n**) or sustained outward current (**o**)

We wanted to assess whether iCtx cells could integrate into the adult human cortex after transplantation. For this purpose, we used organotypic slice cultures of hACtx obtained from epileptic patients undergoing hippocampal resection surgery. We initially evaluated the viability of the hACtx slices before (Fig. 7a–d) and after (Fig. 7e–o) they had been kept in culture for 4 to 6 weeks. Before culturing, cells in hACtx slices exhibited good morphology expressing neuronal and glial markers (Fig. 7a–d). Immunohistochemistry after several weeks of culturing revealed viable tissue with Fox3^+^ and pan-neuronal marker SC121^+^ neurons (Fig. 7e–h). In addition, lentivirus-transduced SynI-RFP^+^ hACtx neurons were able to generate at least one AP (maximum number of APs: 2.6 ± 1.0, *n* = 10), and expressed fast TTX-sensitive inward sodium current and sustained TEA-sensitive outward potassium current (Fig. 7m–o). The cells had an average resting membrane potential of –61.7 ± 4.2 mV, input resistance of 269 ± 46 MΩ, and capacitance of 28.8 ± 7.0 pF. Taken together, these findings provide evidence that the slices were viable, mimicking that of the in vivo condition even after extended culturing.

**Fig. 8:**
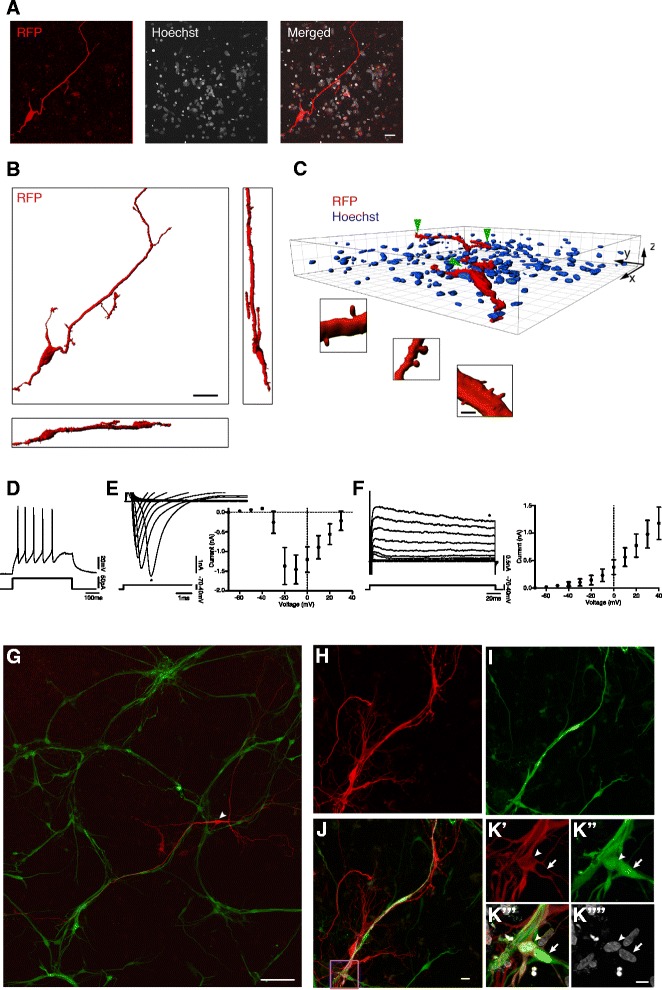
Human iCtx cells integrate into human cortical tissue and form synaptic connections. **a** Confocal images of a SynI-RFP^+^ iCtx cell (*RFP*), induced by BMF and plated on hACtx organotypic slice for 4 weeks, with Hoechst staining of surrounding nuclei. *Scale bar* = 30 μm. **b** Orthogonal views of the three-dimensional reconstruction of the SynI-RFP^+^ iCtx cell in **a**. *Scale bar* = 40 μm. **c** Perspective view of the three-dimensional reconstruction of the SynI-RFP^+^ iCtx cell in **a**. Enlarged images of the spines (*green arrowheads*) in the neurites of the iCtx cell. *Scale bar* = 3 μm. **d** Voltage trace illustrating AP generation during a 50pA current step lasting 500 ms. **e,f** Current traces of the fast inward sodium current (**e**, *left*) and sustained outward potassium current (**f**, *left*) activated by step depolarizations from a holding potential of +70 mV. * Inward current peak (**e**) or sustained outward current (**f**). Current-voltage plot of the fast inward (**e**, *right*) and the sustained outward current (**f**, *right*) (*n* = 10). **g–k** Afferent synapsis on transplanted iCtx cells from host neurons in hACtx slices. **g** Confocal image of hACtx organotypic slice showing an overview of hACtx cell labeling with tetO-GFP and subsequent tracing with ΔG-rabies virus (*white arrowhead*). **h–k** hACtx cells labeled with tetO-GFP (**i**) and retrogradely traced with ΔG-rabies virus (**h**) after infection of grafted iCtx cells were either mCherry /GFP (h) or mCherry /GFP (**k**''', *white arrowhead*). Nontraced hACtx cells were GFP only (**k**''', *white arrow*). **j** Merged image of h and **i**; **k** Magnification of the box in **j** to **k**' (RFP), **k**'' (GFP), **k**''' (merged) and **k**'''' (Hoechst). Scale bar= 100 μm for **g**; 20 μm for **h–j**; 10 μm for **k**

For ex vivo transplantation of iCtx cells, we first cultured the cortical slices for up to 2 weeks. From 10 dpi, the miR124.T-GFP/SynI-RFP-infected iCtx cells were co-cultured on the cortical slices for an additional 3 weeks. Immunohistochemistry and three-dimensional reconstruction of the RFP^+^ cells in the slices revealed that the iCtx cells had survived, integrated morphologically, and extended neurites throughout the adult human cortical tissue (Fig. 8a–c; Additional file 2). The neurites contained a variety of spines with different size and shape, including mushroom-like spines. Moreover, whole-cell patch-clamp recordings revealed that the grafted iCtx cells were functional as they expressed both a fast inward sodium current (Fig. 8e), a sustained outward potassium current (Fig. 8f), and had the capability to generate APs (maximal number of APs: 5.9 ± 2.0, *n* = 10; Fig. 8d). The cells had a resting membrane potential of –29.7 ± 3.2 mV, an input resistance of 512 ± 81 MΩ, and a capacitance of 6.3 ± 1.3 pF (*n* = 10 cells recorded in seven organotypic slices, collected from two patients).

Finally, we wanted to explore if the transplanted iCtx cells could receive direct synaptic input from host neurons in the organotypic slice cultures. For this purpose, we used retrograde monosynaptic tracing by a modified rabies virus (ΔG-rabies) [30, 31]. We first assessed synaptic connectivity between host neurons in non-grafted slice cultures. After a few days in culture, the organotypic slices were transduced to stably express avian TVA receptor (to allow for infection with rabies virus), GP (to allow for spread of virus trans-synaptically), and nuclear GFP (GFP_nuc_; to identify transduced cells). After 5 to 6 weeks, the slice cultures were infected with ΔG-rabies virus carrying SynI-mCherry gene, and 3 days later fixed and immunostained. A number of mCherry^+^ neurons were found to be spread throughout the slice cultures (Fig. 7i–l), the vast majority of them co-expressing GFP_nuc_, which showed that they had been transduced with rabies virus. Importantly, we also observed a few mCherry^+^/GFP_nuc_^–^ cells (Fig. 7l), which indicated that non-transduced neurons in the cultures had been retrogradely labeled due to the selective propagation of rabies virus across active synapses [32]. These findings provide evidence for the occurrence of synaptic connections between adult neurons in the organotypic slice cultures, supporting their viability at 6 weeks.

We then went on to determine if the host neurons in the organotypic cultures could establish afferent synapses also on the grafted iCtx cells. To visualize host cells, we first transfected hACtx slices with FUW-TetO-GFP lentivirus. After a few days, a vast majority of cells in the slices, including many neurons, were expressing cytoplasmic GFP (GFP_cyto_; Fig. 8g). On the organotypic slices, we then plated iCtx cells, which had been transduced to express TVA receptor and GP as described above. Three weeks after ex vivo transplantation, iCtx cells were selectively infected with ΔG-rabies virus carrying SynI-mCherry gene, and 3 days later fixed and immunostained. We found many mCherry^+^/GFP_cyto_^+^ (Fig. 8h–k) host neurons and mCherry^+^/GFP_cyto_^–^ neurons that could be either host or iCtx cells (Fig. 8g). This supports that both these groups of neurons had formed synaptic inputs on iCtx cells as evidenced by the selective spread of mCherry-carrying ΔG-rabies. Taken together, our findings indicate that adult human cortical neurons form afferent synapses on the ex vivo transplanted human iCtx cells.

### Discussion

Here we demonstrate that forced expression of a combination of the transcription factors BRN2, MYT1L, and FEZF2 (BMF) directly converts human fibroblasts to pyramidal-shaped neurons with mature functional properties and extensive arborizations, expressing markers of cortical projection neurons. Single-cell analysis revealed that a subpopulation of these BMF-induced iCtx cells had a gene expression profile similar to that of human fetal primary cortical neurons. Importantly, the BMF-induced iCtx cells were shown to integrate, morphologically and functionally, into human fetal and adult cortical neuronal networks.

Several studies have reported the generation of excitatory (VGLUT1^+^) neurons as a subpopulation of directly converted cells independently of the reprogramming cocktail [2, 12, 25, 33]. In addition, Vierbuchen and co-workers [2] reported that most of the mouse fibroblast-derived iN cells induced by Ascl1, Brn2, and Myt11 (ABM) expressed TBR1, which is a marker of layer VI cortical projection neurons. However, TBR1^+^ glutamatergic neurons are also observed in other brain areas, e.g., the cerebellum [34], and it is unclear if the VGLUT1^+^ iN cells induced by the ABM combination represent a subtype of excitatory neurons. In order to specifically generate cortical neurons, we chose transcription factors based on current knowledge of cortical development. We determined the morphological, functional, and molecular signature of our directly converted cells instead of relying mainly on conversion efficiency and functionality data as performed in previous studies. All tested combinations of transcription factors produced MAP2^+^ iN cells but the most promising one for generating cortical pyramidal neurons was BMF. Interestingly, BMF generated iN cells in the absence of ASCL1, which has been reported to initiate and be sufficient for the direct reprogramming path [35, 36]. The absence of ASCL1 in the BMF combination could explain the low conversion efficiency induced by BMF unless we added small molecules and microRNAs to the reprogramming cocktail.

The BMF combination used here to produce iCtx cells differs from ABM [2] with respect to ASCL1 and one transcription factor, namely FEZF2. This factor is critical for deep-layer cortical neuron development, especially corticospinal motor neurons [37–40]. It has also been shown to change the fate of layer IV neurons to layer V neurons [41, 42], subventricular zone neural stem cells to pyramidal neurons [43], and striatal progenitors to cortical neurons [40]. In addition, *Fezf2* ^*+*^ progenitor cells can give rise to all major types of layer V projection neurons in both layer VA and layer VB of the mature mouse motor cortex [44]. Thus, FEZF2 is a key transcription factor that could potentially determine the phenotype of the precursor cells and override the default program in order to generate cortical pyramidal neurons, especially of the deep-layer subtype.

Interestingly, we observed that some BMF-induced iCtx cells expressed both the SATB2 gene and protein. This callosal projection neuronal marker has been shown to be critical for the development of cortico-spinal motor neurons [45]. In addition, the mutual regulation between SATB2 and FEZF2 enables SATB2 to promote subcerebral neuron identity in layer V neurons, and to repress subcerebral features in callosal neurons [46]. This suggests that SATB2 promotes the development of callosal and subcerebral neurons in a cell context-dependent manner.

Of crucial importance when attempting to produce a specific subtype of neurons by direct conversion of human somatic cells is to determine the composition of the generated cell population and to what extent these cells resemble their in vivo counterparts. In our study, single-cell analysis revealed that BMF gave rise to a heterogeneous population including both non-reprogrammed and partially reprogrammed cells. Most importantly, though, we found that 13.1% of the iCtx cells exhibited a gene expression pattern resembling that of developing human fetal cortical neurons (hCtx cells). Interestingly, Handel and colleagues showed that this is also the case for iPS-derived cortical neurons at the single-cell level [47]. The challenge now is to develop protocols to produce specific subtypes of cortical neurons in more homogeneous populations, which is difficult mainly because of the current lack of unique molecular markers for layer and region. However, this may be soon feasible considering recent advances in mouse [48, 49] and human [50] cortical cell taxonomy.

In order for the iCtx cells to be useful for transplantation as well as for disease modeling, it should be possible to produce them in large numbers. Importantly, we demonstrate that the BMF-induced direct conversion of human fibroblasts to cortical neurons can be substantially scaled up by adding miR-124 and miR-9 along with small molecules to the reprogramming cocktail. Our finding is in line with previous observations that microRNAs synergize with neuron-specific transcription factors to promote neurogenesis [23] and that addition of small molecules does not alter the initial phenotype of iN cells [13].

It is also highly warranted to explore whether the iCtx cells can survive transplantation and integrate into host neuronal circuitries. Previous studies have demonstrated that both mouse and human fibroblast-derived iN cells can survive intracerebral transplantation and integrate into the host brain of mice and rats [11, 13, 51–53]. We provide here the first evidence that transplanted iN cells can survive and integrate also in the human brain. Our findings demonstrate the utility of organotypic slice cultures prepared from adult human cortical tissue [54] in the preclinical assessment of human-derived iN cells. The transplanted iCtx cells survived and extended neurites into the organotypic slice cultures of adult human cortex. The iCtx cells also received afferent synaptic inputs from hACtx cells, which were trans-synaptically labeled by rabies virus. The neurites of the iCtx cells in the organotypic slice cultures had spines similar to those associated with functional synapses in co-cultures of iCtx cells with human fetal hCtx cells.

### Conclusions

Here we demonstrated for the first time that functional neurons with cortical projection characteristics can be generated by direct conversion of human fibroblasts. Our protocol provides new possibilities to produce cells for modeling neurodegenerative disorders that affect cortical neurons. Importantly, we demonstrate survival and integration of the directly converted cortical neurons into adult human cortical tissue after ex vivo transplantation onto organotypic cultures. It will now be important to explore whether human iCtx cells can survive intracortical transplantation in animal models of diseases affecting cerebral cortex, become integrated, and ameliorate functional impairments.

## Additional files

Additional file 1: List of primers/probes used in qPCR analysis. (DOCX 91 kb)

Additional file 2: Three-dimensional video reconstruction of iCtx cell ex vivo transplanted onto adult human-derived cortical tissue. Fly-through animation of three-dimensional data illustrating pyramidal shape of iCtx cell (in red) with elaborated neurites extending throughout the whole depth of the adult human cortical slice at 3 weeks after ex vivo transplantation. Note many spines of different size and shape including mushroom-like ones. Many Hoechst-stained cell nuclei of human cortex (in blue) are distributed throughout the slice. Scale bar in left bottom corner. (MOV 16146 kb)

## Abbreviations

AP: Action potential
dpi: Days postinduction
EPSC: Excitatory postsynaptic current
GFP: Green fluorescent protein
hACtx: Human adult cerebral cortex
hCtx: Human fetal primary cortical
HEFL: Human fetal lung fibroblasts
hStr: Human striatal
iCtx: Induced cortical
iN: Induced neuronal
IPSC: Inhibitory postsynaptic current
PBS: Phosphate-buffered saline
PCA: Principal component analysis
PFA: Paraformaldehyde
PMI: Pyramidal morphology index
Ptx: Picrotoxin
qPCR: Quantitative polymerase chain reaction
RFP: Red fluorescent protein
SM: Small molecule
sPSC: Spontaneous postsynaptic current
TEA: Tetraethylammonium
TTX: Tetrodotoxin
