# Supplementary material for: Direct conversion of human fibroblasts to functional excitatory cortical neurons integrating into human neural networks
Source: Stem Cell Res Ther. 2017 Sep 29;8:207. doi: 10.1186/s13287-017-0658-3 (PMC5622454; doi:10.1186/s13287-017-0658-3)
Supplement: Supplementary file 1 — List of primers/probes used in qPCR analysis. (DOCX 91 kb) [file 13287_2017_658_MOESM1_ESM.docx]

***List of primers/probes used in qPCR analysis.***

| **Target** | **Alias** | **Expression / Function in** | **Assay ID** |
| --- | --- | --- | --- |
| ASCL1 | MASH1 | Diencephalon | Hs04187546_g1 |
| BCL11B | CTIP2 | Deep-layer neurons | HS00256257_m1 |
| BHLHE22 | BHLHB5 | Spatial patterning of neocortex | Hs01084964_s1 |
| CNTN6 |  | Layer 5b cortical-spinal/motor neurons | Hs00274291_m1 |
| COL1A1 | OL4 | Fibroblasts | Hs00164004_m1 |
| CRYM |  | Deep-layer subcerebral projection neurons | Hs00157121_m1 |
| CUX1 | CDP1 | Upper-layer neurons | Hs00738851_m1 |
| CUX2 | CDP2 | Progenitor cells and upper-layer neurons | Hs00390035_m1 |
| DBH |  | Noradrenergic | Hs01089840_m1 |
| DCX |  | Neuroblasts | Hs00167057_m1 |
| DIAPH3 |  | Cortical-spinal/motor neurons | Hs01107330_m1 |
| EMX2 |  | Neural progenitors; Spatial patterning of neocortex | Hs00244574_m1 |
| ETV1 | ER81 | Layer 5 cortico-striatal projection neurons | Hs00951951_m1 |
| FEZF2* |  | Neocortical progenitors; Subcortical projection neurons | Hs01115572_g1 |
| FOXG1 |  | Telencephalon | Hs01850784_s1 |
| FOXO1 |  | Layer 5b & hindbrain | Hs01054576_m1 |
| GAD1 | GAD67 | GABA synthesis | Hs01065893_m1 |
| GAD2 | GAD65 | GABA synthesis | Hs00609534_m1 |
| HTR2C |  | Serotonergic & layer 5 | Hs00168365_m1 |
| LBX1 |  | Hindbrain | Hs00198080_m1 |
| LMO4 |  | Layers 2/3 and 5; Callosal projection neurons | Hs01086790_m1 |
| MAPT | Tau | Pan-neuronal | Hs00902194_m1 |
| MYT1L* |  | Pan-neuronal | Hs00903951_m1 |
| NEUROD1 |  | Telencephalon | Hs00159598_m1 |
| NEUROG2 |  | Neural progenitors | Hs00702774_s1 |
| NR2F1 | COUPTF1 | Temporal lobe (8-12 pcw) | Hs01354342_mH |
| NR4A2 | NURR1 | Dopaminergic/midbrain | Hs00428691_m1 |
| OTX1 |  | Cortical-spinal/motor neurons | Hs00951099_m1 |
| PAX6 |  | Neural progenitors | Hs00240871_m1 |
| PCP4 |  | Layer 5b cortical-spinal/motor neurons | Hs01113638_m1 |
| POU3F2* | BRN2 | Pogenitor cells and upper-layer neurons | Hs00271595_s1 |
| RORB |  | Layer 4 | Hs00199445_m1 |
| S100A10 |  | Layer 5 | Hs00741221_m1 |
| SATB2 |  | Callosal projection neurons; Neocortex | Hs01546828_m1 |
| SLC17A7 | vGLUT1 | Glutamate transport | Hs00220404_m1 |
| SLC17A6 | vGLUT2 | glutamate transport | Hs00220439_m1 |
| SOX1 |  | neural progenitors | Hs01057642_s1 |
| SOX5 |  | cortical-spinal/motor neurons | Hs00753050_s1 |
| SP8 | BTD | neural progenitors | Hs01941366_s1 |
| TBP |  |  | Hs00427621_m1 |
| TBR1 |  | Layer 1 and deep-layer neurons; Telencephalon (8-12 pcw) | Hs00232429_m1 |
| TH |  | Dopaminergic/midbrain | Hs00165941_m1 |
| TLE4 |  | Deep-layer & cortical-thalamic projection neurons | Hs00419101_m1 |
| TPH1 |  | serotonergic | Hs00188220_m1 |
| TUBB3 |  | pan-neuronal | Hs00964963_g1 |
| UBC |  | control | Hs00824723_m1 |
| YWHAZ |  | control | Hs03044281_g1 |

*pcw – post conception week*

** These primers/probes detect endogenous gene expression as well as transgene expression.*
